# *Smilax aristolochiifolia* Root Extract and Its Compounds Chlorogenic Acid and Astilbin Inhibit the Activity of *α*-Amylase and *α*-Glucosidase Enzymes

**DOI:** 10.1155/2018/6247306

**Published:** 2018-06-25

**Authors:** Viridiana Candelaria Pérez-Nájera, Janet Alejandra Gutiérrez-Uribe, Marilena Antunes-Ricardo, Sergio Hidalgo-Figueroa, Carmen Lizette Del-Toro-Sánchez, Luis A. Salazar-Olivo, Eugenia Lugo-Cervantes

**Affiliations:** ^1^División de Desarrollo Biotecnológico, Centro Universitario de la Ciénega-Universidad de Guadalajara, 47820 Ocotlán, Mexico; ^2^Tecnológico de Monterrey, Centro de Biotecnología-FEMSA, 64849 Monterrey, Mexico; ^3^Cátedra CONACYT, IPICYT/Consorcio de Investigación, Innovación y Desarrollo para las Zonas Áridas, 78216 San Luis Potosí, Mexico; ^4^Departamento de Investigación y Posgrado en Alimentos, Universidad de Sonora, 83000 Hermosillo, Mexico; ^5^División de Biología Molecular, Instituto Potosino de Investigación Científica y Tecnológica (IPICYT), 78216 San Luis Potosí, Mexico; ^6^Unidad de Tecnología Alimentaria, Centro de Investigación y Asistencia en Tecnología y Diseño del Estado de Jalisco, 44270 Guadalajara, Mexico

## Abstract

Regulating activities of *α*-amylase and *α*-glucosidase through the use of specific inhibitors is a main strategy for controlling type 2 diabetes.* Smilax aristolochiifolia *root decoctions are traditionally used in Mexico as hypoglycemic and for weight loss, but the active principles and mechanisms underlying such putative metabolic effects are yet unknown. Here, we isolated the major bioactive compounds from a hydroethanolic extract of* S. aristolochiifolia *root by fast centrifugal partition chromatography and evaluated their effects against pancreatic *α*-amylase and yeast *α*-glucosidase. A chlorogenic acid-rich fraction (CAF) inhibited *α*-amylase activity with an IC_50_ value of 59.28 *μ*g/mL in an uncompetitive manner and *α*-glucosidase activity with an IC_50_ value of 9.27 *μ*g/mL in a noncompetitive mode. Also, an astilbin-rich fraction (ABF) inhibited *α*-glucosidase activity with an IC_50_ value of 12.30 *μ*g/mL, in a noncompetitive manner. CAF inhibition *α*-amylase was as active as acarbose while both CAF and ABF were 50-fold more potent inhibitors of *α*-glucosidase than acarbose. The molecular docking results of chlorogenic acid and astilbin with *α*-amylase and *α*-glucosidase enzymes correlated with the inhibition mechanisms suggested by enzymatic assays. Our results prove that* S. aristolochiifolia *roots contain chlorogenic acid and astilbin, which inhibit carbohydrates-hydrolyzing enzymes, suggesting a new mechanism for the hypoglycemic effect reported for this plant.

## 1. Introduction

Diabetes mellitus is one of the most common chronic diseases in nearly all countries and continues to increase in number and significance, as economic development and urbanization lead to lifestyles characterized by reduced physical activity and increased obesity [1]. Diabetes mellitus is characterized by abnormally high plasma glucose concentration, resulting from insufficient or inefficient insulin secretion, with alterations in carbohydrate, protein, and lipid metabolism. Hyperglycemia has played a central role in the pathogenesis of complications related to diabetes mellitus, such as retinopathy, cataract, atherosclerosis, neuropathy, nephropathy, and impaired wound healing [2]. One therapeutic approach for decreasing postprandial hyperglycemia is to reduce the intestinal absorption of glucose from food, through inhibiting the intestinal carbohydrate-hydrolyzing enzymes, *α*-amylase and *α*-glucosidase. Synthetic drugs such as acarbose, voglibose, and miglitol are widely used as inhibitors of these enzymes in the management of patients with type 2 diabetes [3, 4]. However, these inhibitors are reported to cause several side effects, such as abdominal distension, flatulence, meteorism, and diarrhea. Previously studies suggested that the consumption of dietary polyphenols might reduce the risk of type 2 diabetes and its complications [5–8]. Therefore, efforts have been directed toward finding natural and safer *α*-amylase and *α*-glucosidase inhibitors, and the search of such agents in traditional medicinal plants has become more important [9].

*Smilax aristolochiifolia* Miller (Smilacaceae), popularly known as zarzaparrilla, is widely distributed in Mexico [10] and commonly employed as root decoctions indicated as hypoglycemic [11] and for weight loss [12]. Pharmacological research has reported hematopoietic [13], hypoglycemic, and hypotensive effects [14] for the root of* S. aristolochiifolia*. Although antidiabetic potential has also been reported for other* Smilax *species, mainly of* S. china* [15, 16], the identity of bioactive compounds responsible for the antidiabetic effects of* S. aristolochiifolia* as well as their mechanisms of action are yet unknown. Therefore, we aim to identify the major bioactive compounds from* S. aristolochiifolia* root and to characterize their effects on *α*-amylase and *α*-glucosidase enzymatic activities.

## 2. Materials and Methods

### 2.1. Materials

Plants of* Smilax aristolochiifolia* Miller (including the roots) were collected in Apazapan, Veracruz, Mexico (19°19′25.6^″^N and 96°43′17.3^″^W) in October 2015. Plant material was authenticated by Dr. M. Chazaro (Biology Department, Universidad Veracruzana), and a voucher specimen (10855) was deposited in the Institute of Ecology herbarium (IE-XAL), Xalapa, Veracruz, Mexico. *α*-Glucosidase (EC 3.2.1.20, from* Saccharomyces cerevisiae*, 28 U/mg), acarbose, *ρ*-nitrophenyl-*α*-D-glucopyranoside (pNPG), porcine pancreatic *α*-amylase (EC 3.2.1.1, type VI-B, from porcine pancreas, ≥10 U/mg), and 3,5-dinitrosalicylic acid reagent (DNS) were purchased from Sigma-Aldrich Co. (St. Louis, MO, USA). The soluble starch was purchased from Jalmek Científica (Monterrey, NL, Mexico).

### 2.2. Preparation of S. aristolochiifolia Root Extract

The root of the plant was dried in the dark at room temperature and the dried material was then milled with a ball mill. Preliminary assays showed that extraction of* S. aristolochiifolia* roots by aqueous infusion or hydroethanolic maceration gives rise to the same profile of elution (Figure S1), although maceration produced a 2-fold higher yield than infusion (15.28% by infusion and 30.11% by maceration). The extraction was performed by maceration at room temperature (25°C) and stirring overnight using a solid: liquid ratio of 1 : 20 w/v in ethanol: water (1 : 1, v/v) as solvent. The* S. aristolochiifolia *root extract (SAR) was obtained by filtration across Whatman paper no. 4. Then, ethanol was eliminated by concentration under vacuum (IKA RV 10 digital, Staufen, Germany) at 40°C and water by freeze-drying. Dry SAR was stored at −80°C until use.

### 2.3. Fast Centrifugal Partition Chromatography

Fractions from SAR were obtained in a preparative fast centrifugal partition chromatography (FCPC) instrument (Kromaton, Angers, France), with rotor capacity of 1 L, operated in dual-mode: 0–57 min in descending mode, and 58–120 min in ascending mode at 1000 rpm and a flow rate of 10 mL/min using ethyl acetate: water (1 : 1 v/v) as the two-phase solvent system, according to preliminary assays (Table S1). SAR (10 g) was dissolved in 160 mL of solvent system, filtered, and pumped into the rotor. One hundred and twenty fractions were collected and grouped in pools of 10 fractions according to similarity of their partition coefficient (*k*_*d*_) values to facilitate their analysis. A total 12 pools were concentrated to dryness at 45°C under reduced pressure (EZ-2 Plus, Genevac Ltd., UK) and stored at −20°C until testing.

### 2.4. High Performance Liquid Chromatography Analysis

SAR and its FCPC-obtained fractions were analyzed by HPLC-DAD (Agilent Technologies, 1200 Series, Santa Clara, CA) according to the method described by Becerra-Moreno et al. [17] with some modifications. The compounds were separated in a Luna 5 U C_18_, 4.6 mm ID × 250 mm (5 *μ*m) column (Phenomex, Torrance, CA). The mobile phase was constituted by solvent A, HPLC grade water (BDH, Poole, UK) acidified with 0.1% formic acid (CTR Scientific, Monterrey, NL, Mexico), and solvent B, HPLC grade methanol (BDH, Poole, UK), using a gradient at a flow rate of 0.8 mL/min. The proportion of the mobile phase was maintained as follows: 0–3 min (B, 0% to 18%); 3–8 min (B, 18% to 30%); 8–35 min (B, 30% to 42%); 35–40 min (B, 42% to 48%); 40–45 min (B, 48% to 60%); 45–50 min (B, 60% to 100%); 50–60 min (B, 100% to 0%). Chromatograms were obtained at 280 nm, 10 *μ*L of sample was injected, and UV absorption spectra were collected. The results of quantification were expressed as chlorogenic acid or kaempferol-3-*O*-glucoside equivalents, based on the calibration curve of the corresponding standards.

Identification of major compounds was carried out by liquid chromatography coupled with time-of-flight mass spectrometry (LC/MS-TOF) (1100 Series, Agilent Technologies, Santa Clara, CA), using the same chromatographic conditions described above. Ionization was carried out using an electrospray ionization source in positive mode (ESI^+^) with the following conditions: range for mass scan covered from *m*/*z* 140 to 1000, nitrogen gas temperature set at 350°C, gas flow rate at 11 L/min, nebulizer pressure at 50 psi, 3500 V capillary voltage, and 50 V in fragmentor. Extracted ion chromatograms were obtained by considering the exact mass of the compound using Analyst QS 1.1 software (Applied Biosystems, Carlsbad, CA).

### 2.5. Enzyme Inhibition Assays and Action Mechanism Study

#### 2.5.1. Assay of *α*-Amylase Activity

The *α*-amylase inhibitory activity of SAR, CAF, and ABF was determined by measuring the reducing power of released oligosaccharide from soluble starch according to the method of Miller [18]. A series of tests at varying concentrations of both substrate and inhibitor were conducted to determine inhibition types. SAR, CAF, and ABF were prepared at concentrations of 1 to 200 *μ*g/mL in 20 mM phosphate buffer with 6.2 mM sodium chloride at pH 6.9. Porcine pancreatic *α*-amylase at 5 U/mL and 1% soluble starch solutions were prepared in the same buffer. All solutions were prepared immediately prior to each test.

Aliquots of an *α*-amylase solution of 500 *μ*L and a sample solution of 500 *μ*L were mixed in a 15 mL Eppendorf tube and incubated at 20°C for 10 min. The reaction was initiated by adding 1 mL of starch solution into the mixture and incubated during 10 min at 20°C. Afterwards, 1 mL of 3,5-dinitrosalicylic acid (DNS) reagent solution was added followed by heating in boiling water for 15 min to develop color. The reaction was stopped by cooling down in ice water. The reaction mixture was diluted with 9 mL of distilled water and the absorbance was read at 540 nm using a spectrophotometer (UV-VIS 6405, JENWAY, UK). Acarbose was used as a positive control and phosphate buffer as a negative control. The inhibition percentage was calculated following the equation:

(1) $$\begin{array}{c} \%\;\;Inhibition=\frac{{Abs}_{negative\;\;control}-{Abs}_{sample}}{{Abs}_{negative\;\;control}}\times 100 \end{array}$$

#### 2.5.2. Assay of *α*-Glucosidase Activity

The *α*-glucosidase inhibitory activity of SAR, CAF, and ABF was assayed by the pNPG (*ρ*-nitrophenyl-*α*-D-glucopyranoside) method [19]. Briefly, reaction mixtures consisting of 25 *μ*L of *α*-glucosidase from* S. cerevisiae* (0.2 U/mL) (Sigma-Aldrich, G5003) and 25 *μ*L of different concentrations (1 to 200 *μ*g/mL) of sample solutions were preincubated in a 96-well plate at 20°C for 10 min. Afterwards, the reaction was started by adding 50 *μ*L of 2 mM pNPG to each well. After 20 min, the reaction was stopped by adding 50 *μ*L of 0.2 M Na_2_CO_3_. All solutions were prepared immediately prior to each test and 20 mM phosphate buffer with 6.2 mM sodium chloride at pH 6.9 was used as vehicle. The *ρ*-nitrophenol product released from the pNPG substrate was used to quantify the enzymatic activity; the absorbance was measured at 405 nm in a microplate reader (Bio-Rad model 550, Berkeley, CA). The percentage of inhibition was calculated according to (1).

#### 2.5.3. Determination of Enzymatic Inhibitory Model by Kinetics Analysis

The type of enzyme inhibition was graphically determined using the Lineweaver–Burk plot. The inhibition activity (IC_50_) was used to evaluate the effectiveness of an inhibitor. IC_50_ value is defined as the concentration of a test substance required achieving half maximal inhibition of a given reaction. IC_50_ values were calculated using the nonlinear regression and logistic function.

### 2.6. Homology Modeling of *α*-Glucosidase

Unlike *α*-amylase, the crystallographic structure for *α*-glucosidase enzyme is not available; therefore the 3D model of *α*-glucosidase from* Saccharomyces cerevisiae* was generated based on the sequence similarity by using homology modeling. The amino acid sequence of the target protein was retrieved from NCBI (https://www.ncbi.nlm.nih.gov/) with ID: P53341.1. BLASTp server was used against Protein Data Bank database to find the appropriate structure template for the homology model. The alignment between the sequences was performed using the MODELLER v.9.18 program. One hundred models were built and the single model was selected by DOPE (Discrete Optimized Protein Energy) score. The final model was validated using two tools ProSa (Protein Structure Analysis) and QMEAN (Qualitative Model Energy Analysis).

### 2.7. Molecular Docking Studies

Molecular docking studies were used to explore the binding mode between ligand and receptor [20]. According to results of enzymatic assays and inhibition type, we investigated the binding modes of chlorogenic acid and astilbin against *α*-amylase and *α*-glucosidase enzymes. First, the three-dimensional structure of porcine pancreatic *α*-amylase with malto-oligosaccharides (PDB ID: 1UA3) was obtained from the Protein Data Bank (PDB) database. For calculation, the malto-oligosaccharides were conserved and all water molecules were removed from the crystallographic structure. The molecular docking was performed using AutoDock 4.2 and AutoDock Tools (ADT, v.1.5.6). The grid dimensions were adjusted to 60 × 60 × 60 points separated by 0.375 Å. PyMOL (PyMOL Molecular Graphics System, San Carlos, CA, USA) and Discovery Studio Visualizer v.17.2.0.16349 (BIOVIA, San Diego, CA, USA) were used for visualization.

On the other hand, the 3D structures of chlorogenic acid and astilbin were obtained from PubChem in the NCBI database. The dimensions of the grid, which represents the coordinates of the parameters in which the ligand can be moved, were 30 × 40 × 40 points separated by 1.0 Å. To establish the grid, it was chosen based on the binding sites reported in noncompetitive inhibition for *α*-glucosidase, covering the residues ASP214, GLU276 and ASP349, ILE149, PRO150 and ASP232, and SER311, PRO312, VAL319, THR310, GLY309, VAL308, ASP307, PHE321 and PRO320 [21–23]. In the default parameters, the Lamarckian genetic algorithm (LGA) was chosen for docking calculations, and 100 experiments per ligand were performed. Compared with the rigidity of the protein, the ligand remained flexible. In the results of molecular docking, the pose with lowest docking energy and maximum number of conformations was selected to represent its most favorable binding mode predicted by this program.

### 2.8. Statistical Analysis

The enzymatic assays were performed in triplicate. The results were analyzed using Statgraphics Centurion XVII v.17.2.00 with Tukey's HSD test. For each data set, *P* < 0.05 was considered statistically significant. The experimental results were expressed as the mean ± standard deviation of at least two separate experiments.

## 3. Results and Discussion

### 3.1. Analysis of S. aristolochiifolia Root Extract and Isolation of Chlorogenic Acid and Astilbin by Fast Centrifugal Partition Chromatography (FCPC)

**Figure 1:**
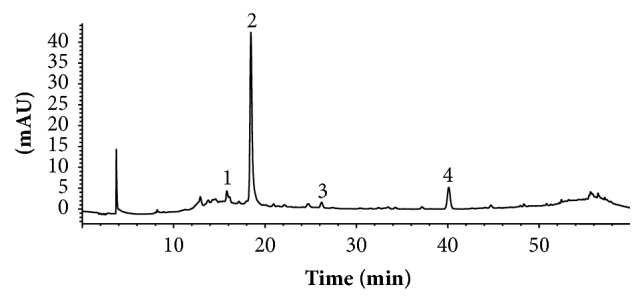
HPLC-UV/Vis chromatogram shown at 280 nm of* S. aristolochiifolia* root hydroethanolic extract. Conditions: reverse-phase C18 column (4.6 × 150 mm, 5 *μ*m, Phenomex); mobile phase, water acidified with 0.1% formic acid and methanol using a concentration gradient (see methodology); flow rate: 0.8 mL/min.

**Figure 2:**
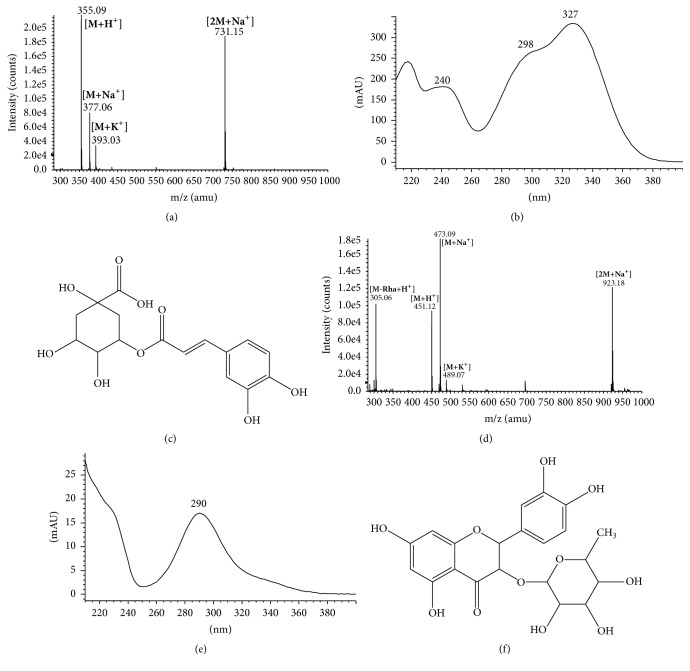
Tentative identification of chlorogenic acid (c) and astilbin (f) by spectral analyses of mass spectrum by ESI/MS (a, d) and UV-vis (b, e).

Chromatographic analyses of SAR at 280 nm (Figure 1) showed two main phenolic constituents, peak 2 eluting at 18.59 min and peak 4 eluting at 40.37 min. Two minor SAR constituents, peaks 1 and 3, were not considered in the present work. Peaks 2 and 4 were tentatively identified according to their UV absorption, *m*/*z*, fragmentation patterns, and previously reported data. The UV-vis spectrum of peak 2 showed characteristic bands of a caffeine residue with *λ*_max_ of 240 and 327 nm (Figure 2(b)) and a molecular ion of 355.09 *m*/*z* [M + H^+^] (Figure 2(a)) corresponding to the chlorogenic acid (Figure 2(c)). The identity of chlorogenic acid was corroborated by standard retention time (data not showed). The UV-vis spectrum of peak 4 had an absorption maximum of 290 nm (Figure 2(e)) and a molecular ion of 451.12 *m*/*z* [M + H^+^] (Figure 2(d)), which is characteristic of astilbin, a flavonoid compound (Figure 2(f)) [24]. Our results constitute the first report of the presence of chlorogenic acid and astilbin in* S. aristolochiifolia*, though both compounds have been previously reported for other species in the genus* Smilax* [25–30].

Chlorogenic acid is a phenolic compound with important pharmacological properties, such as neuroprotective [31], antihyperlipidemic [32], hypoglycemic [33, 34], insulin secretagogue, and sensitizer [35–37]. Chlorogenic acid is present in many plant species and appreciable chlorogenic acid contents have been shown in* Cecropia obtusifolia*,* Vaccinium corymbosum*,* Ilex paraguariensis*,* Camellia sinensis*, and green coffee beans [34, 38–40]. In this study, 83.19 mg chlorogenic acid equivalents per gram of SAR (2504 mg/100 g dry matter) were obtained (Table 1). This concentration of chlorogenic acid is higher than those reported for other sources such as* I. paraguariensis* (1599.6 mg/100 g dry matter) [38],* Cecropia obtusifolia* (1330 mg/100 g dry matter) [34], and coffee pulp (309.7 mg/100 g dry matter) [41] and makes* S. aristolochiifolia* an advantageous source of chlorogenic acid. On the other hand, 3.72 mg of astilbin expressed as kaempferol-3-*O*-glucoside equivalents was obtained per gram of SAR (112 mg/100 g dry matter) below other species as* Smilax glabra* (1%–4%, w/w) [42] or* Engelhardia roxburghiana* [43] (Table 1).

**Figure 3:**
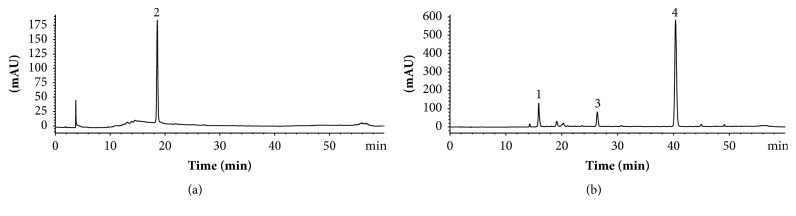
HPLC UV/Vis chromatograms of chlorogenic acid (CAF; a) and astilbin (ABF; b) rich fractions obtained by preparative FCPC from* S. aristolochiifolia* root extract. Conditions: two-phase solvent system, ethyl acetate: water (1 : 1 v/v); dual-mode: 0–57 min in descending mode and 58–120 min in ascending mode; flow rate, 10 mL/min; rotation speed, 1000 rpm; monitored at 280 nm.

When SAR was subjected to one-step FCPC separation, chlorogenic acid was recovered mainly in fractions around of 0.22 *k*_*d*_ (Figure 3(a)), while astilbin appeared mainly in fractions with a *k*_*d*_ value of 2.68 (Figure 3(b)). It was possible to recover both compounds in one-step FCPC separation because we used the dual-mode in which switching the phases extrudes the contents of the column, retrieving compounds of high *k*_*d*_ values as peak 4. The pool of chlorogenic acid-enriched fractions (CAF) achieved concentrations of this compound up to 1.02-fold more than SAR and the pool of astilbin-enriched fractions (ABF) reached 13.11-fold more astilbin than extract (Table 1). CAF and ABF were selected for the next assays.

### 3.2. Effects of S. aristolochiifolia Root Extract (SAR), Chlorogenic Acid Fraction (CAF), and Astilbin Fraction (ABF) on *α*-Amylase and *α*-Glucosidase Activities

**Figure 4:**
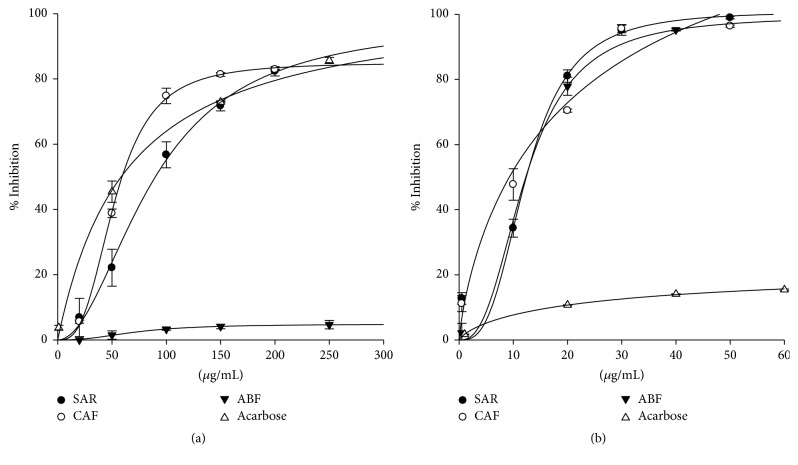
Inhibition of *α*-amylase (a) and *α*-glucosidase (b) enzymes by* S. aristolochiifolia* root (SAR) and its fractions rich in chlorogenic acid (CAF) and astilbin (ABF).

SAR, CAF, and ABF blocked the pancreatic *α*-amylase activity in a concentration-responsive manner. SAR 50 *μ*g/mL inhibited *α*-amylase activity by 22% while SAR 100 *μ*g/mL inhibited *α*-amylase by 56% and SAR 200 *μ*g/mL reached an enzymatic inhibition of 82%, a similar inhibition level as the corresponding acarbose concentration. On the other hand, CAF 50 *μ*g/mL inhibited *α*-amylase activity by 40%, while CAF 100 *μ*g/mL inhibited the enzyme by 75%, similar to acarbose. On the contrary, ABF exerted only marginal inhibitory effects against *α*-amylase at all the assayed concentrations (Figure 4(a)). The IC_50_ values in our *α*-amylase assays were 90.01 ± 3.97 *μ*g/mL for SAR and 59.28 ± 1.30 *μ*g/mL for CAF, this last one statistically similar to the acarbose (IC_50_: 58.59 ± 1.06 *μ*g/mL). Our results confirm recent reports showing the inhibitory effects of chlorogenic acid on *α*-amylase activity [44–46].

We also evaluated the effects of SAR and its fractions on yeast *α*-glucosidase activity. SAR 10 *μ*g/mL inhibited *α*-glucosidase activity by 34% and SAR 20 *μ*g/mL inhibited this enzyme by 81%. In the same manner, CAF 10 *μ*g/mL inhibited *α*-glucosidase activity by 47% and CAF 20 *μ*g/mL inhibited *α*-glucosidase by 70%, while ABF 10 *μ*g/mL blocked *α*-glucosidase in 35% and ABF 20 *μ*g/mL inhibited the enzyme by 78%. All three preparations at 50 *μ*g/mL blocked *α*-glucosidase activity by 98% (Figure 4(b)). The IC_50_ values in our *α*-glucosidase assays were 12.39 ± 0.33 *μ*g/mL for SAR, 9.27 ± 2.05 *μ*g/mL for CAF, and 12.30 ± 0.91 *μ*g/mL for ABF. The inhibition of *α*-glucosidase by* S. china* stem extract and chlorogenic acid has been previously reported but this is the first report on the inhibition of *α*-glucosidase by astilbin. Moreover, the *α*-glucosidase IC_50_ value of SAR was sensibly lower than IC_50_ value reported for* S. china* stem extract (IC_50_: 51.7 *μ*g/mL) [15], suggesting that* S. aristolochiifolia* could be an optimal source for inhibitors of *α*-glucosidase among* Smilax* species. CAF had the highest inhibitory activity compared with other preparations; these results are in accordance with a strong inhibition of chlorogenic acid against *α*-glucosidase [44, 47]. SAR and its FCPC-obtained fractions had up to 50-fold more effective *α*-glucosidase inhibitory activity compared with acarbose (IC_50_: 673.29 ± 53.04 *μ*g/mL). Therefore, the inhibitory activity of* S. aristolochiifolia root* extract and its FCPC-obtained fractions against pancreatic *α*-amylase and yeast *α*-glucosidase may be one of the mechanisms of the hypoglycemic effect of* S. aristolochiifolia *[14]. However, further studies are required to extend this effect to mammalian systems.

### 3.3. Enzymatic Inhibitory Model

**Figure 5:**
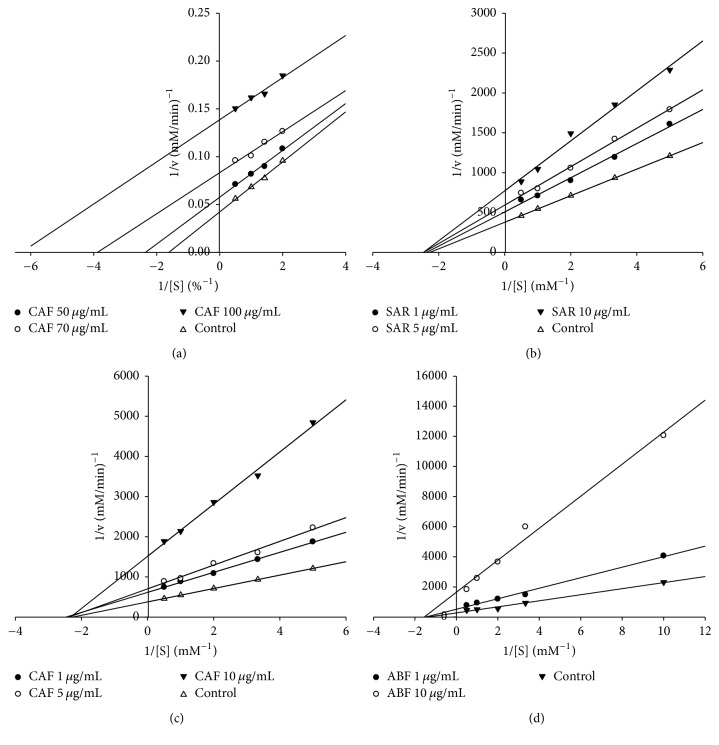
Lineweaver-Burk plots of *α*-amylase (a) and *α*-glucosidase (b, c, and d) activities by* S. aristolochiifolia* root extract (SAR), chlorogenic acid-rich fraction (CAF), and astilbin-rich fraction (ABF). The kinetics was assayed in the absence (control) and the presence of three different concentrations of each preparation.

The double reciprocal Lineweaver–Burk plots revealed that the CAF inhibition of *α*-amylase was uncompetitive since *k*_*m*_ and *v*_max⁡_ values were affected at the same degree by different CAF concentrations (Figure 5(a)). On the other hand, the inhibition of *α*-glucosidase by SAR, CAF, and ABF preparations was noncompetitive since *k*_*m*_ values for different concentrations of these preparations remained constant while their *v*_max⁡_ values decreased with increased inhibitor concentration (Figures 5(b), 5(c), and 5(d)). Noncompetitive inhibition of *α*-glucosidase has been reported for chlorogenic acid [48], apigenin [23], and xanthone derivatives [22].

### 3.4. Three-Dimensional Structure of *α*-Glucosidase Obtained from Homology Modeling

**Figure 6:**
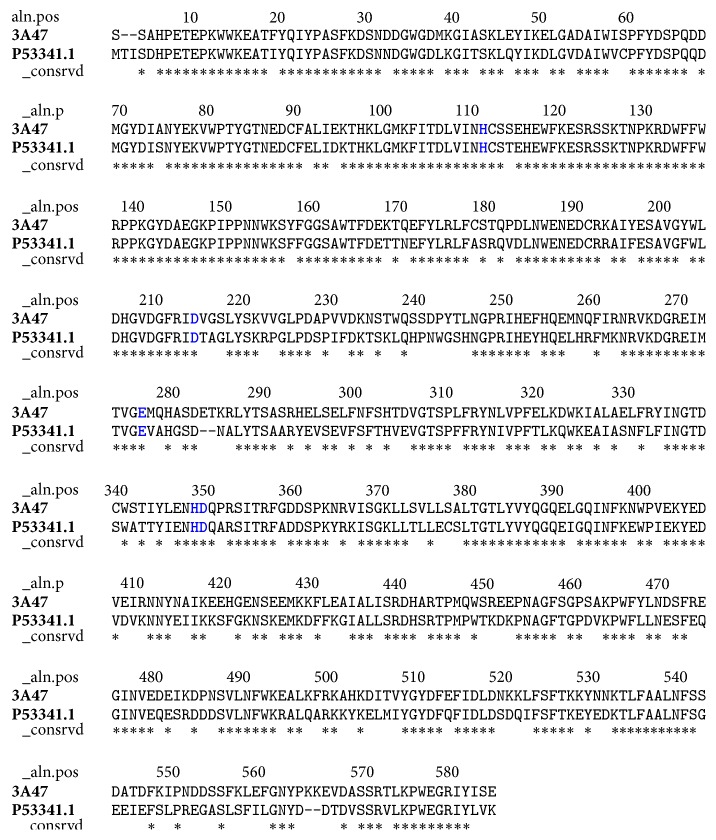
Sequence alignment between *α*-glucosidase (MAL12) (NCBI ID: P53341.1) and isomaltase (PDB ID: 3A47). *∗* indicates conserved residues. The active site residues are indicated in bold blue types.

**Figure 7:**
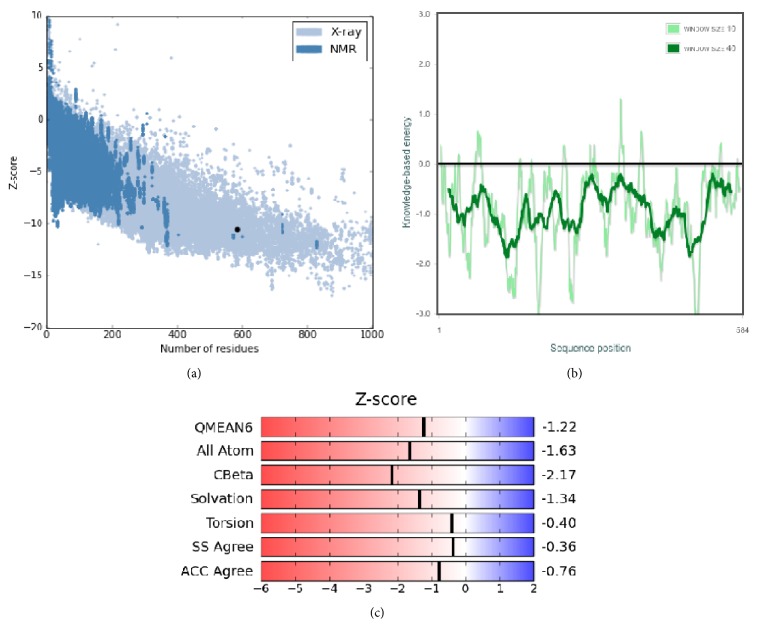
Model evaluation in function of QMEAN Z-score (a, c) and comparison of the ProSa energy profiles for the homology modeled structure of *α*-glucosidase (light green) and the X-ray structure of isomaltase (green) (b).

Alignment analysis with Blastp showed that isomaltase from* Saccharomyces cerevisiae* has a high sequence identity (72%) with the target; besides the active site is highly conserved (Figure 6). Therefore, the X-ray crystal structure of isomaltase from* S. cerevisiae* (PDB ID: 3A47) was selected as template for homology modeling. According to the validation analysis, the theoretical model for *α*-glucosidase was within the range of scores typically found for native proteins of similar size (Figure 7(a)). Furthermore, a QMEAN *Z* score value of −1.22 for the predicted *α*-glucosidase indicated it was of comparable quality to experimental structures (Figure 7(c)). On the other hand the energies in function of the position of the sequence of amino acids showed negative values, considering that positive values correspond to problematic or erroneous parts of the input structure (Figure 7(b)). Consequently, the predicted model for *α*-glucosidase is a reliable model to performance molecular docking studies.

### 3.5. Molecular Docking Analysis on *α*-Amylase and *α*-Glucosidase

**Figure 8:**
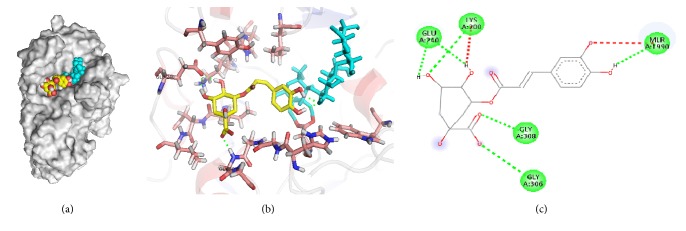
Molecular docking for chlorogenic acid with *α*-amylase (a). Binding uncompetitive mode (b) and two-dimensional interaction diagram (c). Green and red dashed lines show hydrogen bonds and unfavorable donor-donor interactions, respectively. Residues involved in hydrogen bonds (green circles) are shown.

To get further insight into the binding mode between astilbin and chlorogenic acid with *α*-glucosidase and chlorogenic acid with *α*-amylase, the molecular docking was carried out. The docking analysis for chlorogenic acid with *α*-amylase was done on the enzyme-substrate complex and the interactions were predicted close of active site, due to the fact that chlorogenic acid showed an uncompetitive inhibition. Unlike acarbose, which is a strong competitive inhibitor of *α*-amylase, chlorogenic acid requires the formation of enzyme-substrate complex for binding. In the uncompetitive inhibition, the binding of substrate at the catalytic site may modify the *α*-amylase structure, making the inhibitor binding site available. The better pose for chlorogenic acid obtained from docking analysis showed lowest energy (−5.97 Kcal/mol), with highest binding affinity. The main interactions of chlorogenic acid with *α*-amylase involved conventional hydrogen bonds with residues LYS200, GLU240, GLY306, and GLY308, in addition to a malto-oligosaccharide molecule (Figure 8(c)). Our results suggested an important correlation between the uncompetitive inhibition on *α*-amylase (*in vitro* assay) and predicted molecular ligand-enzyme interactions. The hydroxyl groups of chlorogenic acid could be anchored near the active site of *α*-amylase (Figure 8(a)).

**Figure 9:**
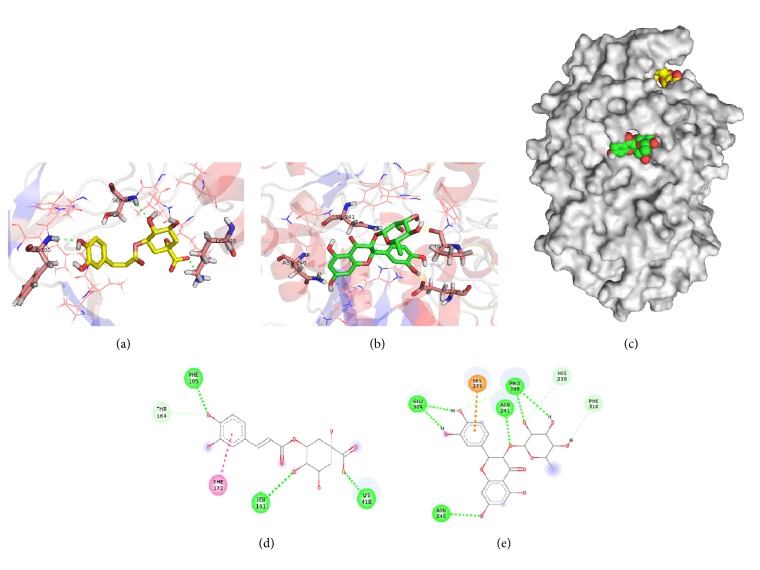
Molecular docking for chlorogenic acid (yellow) and astilbin (green) with *α*-glucosidase (c). Docking of chlorogenic acid (a) and astilbin (b) and corresponding two-dimensional interaction diagrams (d, e). Green dashed lines show hydrogen bonds. Residues involved in hydrogen bonds (green circles), *π*-*π* T-shaped (pink circles), or *π*-cation (orange circles) interactions are shown.

On the other hand, for the analysis of molecular docking for *α*-glucosidase, both compounds had different binding characteristics, as shown in their suggested binding modes (Figure 9). While the binding site for chlorogenic acid on *α*-glucosidase was situated in a place away from the active site, astilbin was close to the active site of the enzyme; both poses corresponded with noncompetitive inhibition mode (Figure 9(c)). The complex of *α*-glucosidase-chlorogenic acid showed lowest binding energy of −3.75 kcal/mol. Chlorogenic acid formed hydrogen bonds with the residues SER161, PHE165, and LYS418, and a *π*-*π* T-shaped interaction between A ring and PHE172 was found (Figure 9(d)). It is likely that this binding interaction of chlorogenic acid to the allosteric site of *α*-glucosidase would probably perturb the protein structure and subsequently the enzyme activity. A similar result was obtained with xanthone derivatives, as noncompetitive inhibitors of *α*-glucosidase with interactions of hydrogen bonding and *π*-*π* stacking [21].

The *α*-glucosidase-astilbin inhibitor complex showed lowest binding energy of −4.56 kcal/mol compared with other poses. Astilbin formed hydrogen-bonding interactions with residues GLU304, PRO309, ASN241, and ASN246, and a *π*-cation interaction between B ring with HIS279 was found (Figure 9(e)). The hydrogen bonds played a role in the binding of astilbin to *α*-glucosidase; two hydrogen bonds were formed between the oxygen of the hydroxyl groups at the C-3′ and C-4′ position on the B ring of astilbin and GLU304. Although the glycosylation of flavonoids decreased the inhibition on *α*-glucosidase, compared to quercetin, astilbin formed hydrogen bonds between hydroxyl groups of rhamnosyl moiety and HIS239, PRO309, and PHE310 [49]. Flavonoids like apigenin presented noncompetitive inhibition against *α*-glucosidase, its binding site is very similar to astilbin, and the interaction with residues close to active site might induce channel closure to prevent access of the substrate [23]. Previous studies have shown that the number of hydrogen bonds had no effect on binding affinity; the hydroxylation of flavonoids improved the inhibitory effects against *α*-glucosidase, which might cause conformational changes in the enzyme structure. In both molecular docking analyses, hydrogen bonds had an important role in biological recognition process and implication of *π*-*π* stacking interaction might be the origin of the enhanced inhibitory activities [5].

## 4. Conclusions

This is the first report on the presence of chlorogenic acid and astilbin in the roots of* Smilax aristolochiifolia* and on the inhibition of *α*-glucosidase by astilbin. Our results indicate that these compounds exert noncompetitive inhibition through two different binding sites on *α*-glucosidase. Moreover, chlorogenic acid shows an efficient* in vitro* uncompetitive inhibition against *α*-amylase. Our results suggested that inhibition of *α*-glucosidase and *α*-amylase by* Smilax aristolochiifolia* and its compounds chlorogenic acid and astilbin could be one of the mechanisms of the hypoglycemic properties attributed to this plant. However, their antidiabetic potential need be corroborated on* in vivo* assays of postprandial hyperglycemia.

## Figures and Tables

**Table 1:** Chlorogenic acid and astilbin contents in SAR, CAF, and ABF.

Sample	*k*_*d*_	Chlorogenic acid content (mg CAE/g)	Astilbin content (mg KGE/g)
SAR	-	83.19	3.72
CAF	0.22	84.77	ND
ABF	2.68	ND	48.76

CAE, chlorogenic acid equivalents; KGE, kaempferol-3-*O*-glucoside equivalents; ND, not detected. *S. aristolochiifolia* root extract, SAR; chlorogenic acid-rich fraction, CAF; astilbin-rich fraction, ABF.

## Data Availability

The data used to support the findings of this study are available from the corresponding author upon request.

## Supplementary Materials

Supplementary 1 Figure S1: profile of* S. aristolochiifolia* root extract obtained by aqueous infusion (blue) and hydroethanolic maceration (red). HPLC UV/Vis at 280 nm. Extraction conditions for aqueous infusion were 60°C under stirring for 1 hour and hydroethanolic maceration consisted in leaving the sample in ethanol : water (1 : 1 v/v) at room temperature (25°C) and stirring overnight; in both processes, the solid : liquid proportion of 1 : 20 w/v was used.

Supplementary 2 Table S1: *k*_*d*_ values of the major peaks of SAR at different concentrations in two solvents systems.
