# Supplementary material for: Smilax aristolochiifolia Root Extract and Its Compounds Chlorogenic Acid and Astilbin Inhibit the Activity of α-Amylase and α-Glucosidase Enzymes
Source: Evid Based Complement Alternat Med. 2018 Jun 25;2018:6247306. doi: 10.1155/2018/6247306 (PMC6036819; doi:10.1155/2018/6247306)
Supplement: Supplementary 1 — Figure S1: profile of S. aristolochiifolia root extract obtained by aqueous infusion (blue) and hydroethanolic maceration (red). HPLC UV/Vis at 280 nm. Extraction conditions for aqueous infusion were 60°C under stirring for 1 hour and hydroethanolic maceration consisted in leaving the sample in ethanol : water (1 : 1 v/v) at room temperature (25°C) and stirring overnight; in both processes, the solid : liquid proportion of 1 : 20 w/v was used. [file 6247306.f1.docx]

Figure S1. Profile of *S. aristolochiifolia* root extract obtained by aqueous infusion (blue) and hydroethanolic maceration (red). HPLC UV/Vis at 280 nm. Extraction conditions for aqueous infusion were 60°C under stirring for 1 hour and hydroethanolic maceration consisted in leaving the sample in ethanol: water (1:1 v/v) at room temperature (25ºC) and stirring overnight, in both process the solid: liquid proportion of 1:20 w/v was used.
