# Supplementary material for: Smilax aristolochiifolia Root Extract and Its Compounds Chlorogenic Acid and Astilbin Inhibit the Activity of α-Amylase and α-Glucosidase Enzymes
Source: Evid Based Complement Alternat Med. 2018 Jun 25;2018:6247306. doi: 10.1155/2018/6247306 (PMC6036819; doi:10.1155/2018/6247306)
Supplement: Supplementary 2 — Table S1: kd values of the major peaks of SAR at different concentrations in two solvents systems. [file 6247306.f2.docx]

Table S1. k_d_ values of the major peaks of SAR at different concentrations in two solvents systems.

| **Solvent system** | **SAR (mg/mL)** | **Peak number** | | | |
| --- | --- | --- | --- | --- | --- |
|  |  | *1* | *2* | *3* | *4* |
| S_1_ ^a^ | 3.125^a^ | 4.80 ± 0.20 | 0.10 ± 0.01 | NQ | 4.70 ± 0.37 |
|  | 6.25^a^ | 3.16 ± 0.76 | 0.11 ± 0.01 | NQ | 4.49 ± 0.26 |
|  | 12.5^b^ | 29.37 ± 2.96 | 1.04 ± 0.02 | NQ | 26.81 ± 0.27 |
|  | 25.0^b^ | 24.30 ± 1.41 | 1.12 ± 0.25 | 12.9 ± 1.51 | 34.50 ± 1.01 |
| S_2_ ^b^ | 3.125^a^ | 4.65 ± 0.03 | 0.59 ± 0.03 | NQ | NQ |
|  | 6.25^a^ | 3.65 ± 1.89 | 0.64 ± 0.05 | NQ | 18.34 ± 0.81 |
|  | 12.5^b^ | 55.17 ± 3.16 | 6.43 ± 0.19 | NQ | 119.78 ± 0.14 |
|  | 25.0^b^ | 49.28 ± 0.48 | 6.47 ± 0.20 | NQ | 116.15 ± 8.18 |

Peak 1, unknown; peak 2, chlorogenic acid; peak 3, caffeoylshikimic acid; peak 4, astilbin. S_1_, ethyl acetate: water, 1:1 v/v; S_2_, butanol: water, 1:1 v/v. NQ: not quantified, the compound is present in a single phase. Different letters indicate significant differences (*P*<0.05). *S. aristolochiifolia* root extract, SAR.
